# The relationship between adolescents’ well-being and their wireless phone use: a cross-sectional study

**DOI:** 10.1186/1476-069X-12-90

**Published:** 2013-10-22

**Authors:** Mary Redmayne, Euan Smith, Michael J Abramson

**Affiliations:** 1 School of Geography, Environment and Earth Sciences, Victoria University of Wellington, P.O. Box 600, Wellington, New Zealand; 2 Department of Epidemiology and Preventive Medicine, School of Public Health & Preventive Medicine, Monash University, The Alfred, Melbourne, VIC 3004, Australia

**Keywords:** Cellular telephone, Cordless telephone, Headache, Tinnitus, Sleep, Depression, Frequency-specific, Headset

## Abstract

**Background:**

The exposure of young people to radiofrequency electromagnetic fields (RF-EMFs) has increased rapidly in recent years with their increased use of cellphones and use of cordless phones and WiFi. We sought to ascertain associations between New Zealand early-adolescents’ subjective well-being and self-reported use of, or exposure to, wireless telephone and internet technology.

**Methods:**

In this cross-sectional survey, participants completed questionnaires in class about their cellphone and cordless phone use, their self-reported well-being, and possible confounding information such as whether they had had influenza recently or had a television in the bedroom. Parental questionnaires provided data on whether they had WiFi at home and cordless phone ownership and model. Data were analysed with Ordinal Logistic Regression adjusting for common confounders. Odds ratios (OR) and 95% confidence intervals were calculated.

**Results:**

The number and duration of cellphone and cordless phone calls were associated with increased risk of headaches (>6 cellphone calls over 10 minutes weekly, adjusted OR 2.4, CI 1.2-4.8; >15 minutes cordless use daily adjusted OR 1.74, CI 1.1-2.9)). Texting and extended use of wireless phones was related to having a painful ‘texting’ thumb). Using a wired cellphone headset was associated with tinnitus (adjusted OR 1.8, CI 1.0-3.3), while wireless headsets were associated with headache (adjusted OR 2.2, CI 1.1-4.5), feeling down/depressed (adjusted OR 2.0, CI 1.1-3.8), and waking in the night (adjusted OR 2.4, CI 1.2-4.8). Several cordless phone frequencies bands were related to tinnitus, feeling down/depressed and sleepiness at school, while the last of these was also related to modulation. Waking nightly was less likely for those with WiFi at home (adjusted OR 0.7, CI 0.4-0.99). Being woken at night by a cellphone was strongly related to tiredness at school (OR 3.49, CI 1.97-6.2).

**Conclusions:**

There were more statistically significant associations (36%) than could be expected by chance (5%). Several were dose-dependent relationships. To safeguard young people’s well-being, we suggest limiting their use of cellphones and cordless phones to less than 15 minutes daily, and employing a speaker-phone device for longer daily use. We recommend parental measures are taken to prevent young people being woken by their cellphones.

## Background

The exposure of young people to radiofrequencies (RF-EMFs) has increased rapidly in recent years with their increased use of cellphones, use of cordless phones and pervasive presence of WiFi, often both at home and at school. Several official bodies and researchers have expressed caution about possible health outcomes from this increasing exposure to RF-EMFs and the accompanying extra low frequencies resulting from modulation. These concerns are due to the young usually having a higher susceptibility to environmental ‘toxins’ and stressors. Although several countries have issued warnings suggesting reduced use of cellphones by children as a precautionary measure, New Zealand has not followed suit. There are still limited studies of general health and well-being outcomes of young people’s exposure to cellphones, cordless phones, or WiFi.

Health is “a state of complete physical, mental and social well-being, and not merely the absence of disease or infirmity” [1]. One basic requirement for general well-being is sufficient good quality sleep. In a study of children aged 9 and 10 years, owning a mobile phone has been associated with settling to sleep after 9 pm, with a quarter of the age group getting less than the 10 hours’ sleep the authors cite as necessary to maintain children’s good health [2]. Preliminary results of a 4-year longitudinal study of mobile communication use by children aged 7-12 years identified related trends including increased fatigue [3]. A European study found tiredness among teenagers associated with increasing cellphone use after lights out, with odds ratios of 1.8 for use less than once a month to 5.1 for more than once weekly [4]. Fatigue was also reported by participants in a German study of 8-12 year olds which measured all daytime RF-EMFs exposures [5]. In this case no statistically significant correlation was found between RF-EMFs exposure and fatigue or other chronic symptoms, apart from reduced likelihood of having sleeping problems, significant in the 3^rd^ [2^nd^ highest] RF-EMF exposure quartile. Headaches were assessed in 8 of the 17 studies in a recent meta-analysis. The headache studies had a total of 737 participants and found an overall marginal association with RF-EMFs [6]. The standardised mean group difference for headache after exposure compared to no exposure was 0.08 and 95% CI -0.02 to 0.18. This suggested increased headache prevalence of 8%, although this is not quite statistically significant at the 95% level since the confidence interval span across zero (null effect). This inconclusive pooled effect warrants further study. Other symptoms that have been described with RF-EMFs exposure include chronic tinnitus [7] and depression [8].

We asked New Zealand adolescents about several well-being measures. We examined whether there was any association between these disorders and their self-reported use of or exposure to wireless technology. We refer to cellphones and cordless phones collectively as wireless phones.

## Methods

### Participants, setting and research instruments

A cross-sectional survey exploring wireless phone user-habits among adolescents of the Wellington Region, New Zealand, was carried out between mid-June and October 2009. Subjective well-being and lifestyle questions were asked towards the end of the questionnaire. There was the option to provide written comments on the well-being questions/responses. Student questionnaires retrieved information about whether the participant had a computer using wireless broadband near the bed, but as the responses were internally inconsistent they were not used. Student participants also provided information on their cellphone make and model. Further details published elsewhere [9].

Some symptoms measuring well-being were drawn from the WHO *Health Behaviour In School-aged Children* (HBSC) checklist (headache, feeling low, sleeping difficulties) [10].

Parental questionnaires included questions on family ownership of a wired landline phone, the make and model of their current cordless phone (for those who had one), and whether they had WiFi at home. The cordless phone’s make and model data were used to ascertain the frequency range and system of each phone, e.g. 2.4 GHz and DECT, respectively. This information was retrieved by the first author from each model’s manual, available online. Students’ socio-economic status (SES) was taken as being that of the school decile rating.

All participants were allocated numbered questionnaires which were completed during a morning period at school. The first author led each session, giving participants the chance to ask questions if clarification was needed. The class teacher was present. Participants were asked not to discuss their answers with neighbours and were assured that their responses would be confidential.

Exposure variables were self-reported cordless and cellphone use, the use and type of cellphone headset, cordless phone frequency and modulation/system approach, and having a WiFi transmitting device at home. The number and/or duration of phone use for calls were collected as self-reported continuous data.

Well-being variables provided data on whether over the previous month, participants had had trouble falling asleep, been waking up in the night, been tired during school, and whether they had had headaches, been feeling down or depressed, experienced tinnitus, or had had a painful texting thumb.

Students were introduced to the survey’s purpose as wanting to find out mainly about their cellphone and cordless phone user-habits (these results are already published [9,11]). They were also informed that there were extra questions about their recent general level of health and exercise, and their use of television and electronic games. These questions were asked after all phone use questions. This along with a low public awareness of the cellphone risk debate in New Zealand (and virtually none regarding cordless phones) would be expected to have minimised any influence on how participants answered the phone use questions.

Possible confounding influences we considered were age, sex, the socioeconomic rating of the school (SES), having recently had a cold or flu, usual bedtime, exercise levels, weekend viewing/electronic gaming hours, TV in the bedroom, the number of times woken weekly by the cellphone, and cellphone storage and carrying habits. Age, the number of times woken by the phone, and the time of settling to sleep were continuous variables, but also categorised. Socioeconomic rating was grouped into decile 1-3, decile 4-7 and decile 8-10.

### Ethical approval

Ethical approval was given by the Victoria University of Wellington human ethics committee. Students could choose to opt out; they could also decline to answer questions. Informed consent was obtained from principals of participating schools and parents of participating students.

### Statistical methods

Statistical analyses were undertaken using SPSS version 19.0. Associations between outcome variables and possible confounders were first assessed with Pearson Chi square tests. Those with p < 0.1 were included in ordinal logistic regression models used to assess RF-EMF associations; this level was selected at the preliminary stage to ensure not excluding those of marginal significance. Continuous exposure variables were split into quintiles, quartiles or tertiles depending upon the variable in question and the ability to fit a model (see Tables).

Well-being questions asked participants to rate the frequency of various symptoms over the last month. These were the categories of choice: A. no, or hardly ever; B. 1 or 2 times/days/nights weekly; C. 3 or 4 times/days/nights weekly. D. most days/nights, or ‘most of the time’ for tinnitus and experiencing a painful thumb, or ‘yes, all the time’ for feeling down or depressed. Ordinal logistic regression models were fitted for cellphone and cordless phone use, use of wired or wireless cellphone headset, wireless broadband at home, and the frequency and system of the cordless phone. Models were regarded as valid if they met the requirements for the overall -2 log-likelihood Chi-square test (*p* < 0.05), the goodness-of-fit test (*p* > 0.05), and the test of parallelism (*p* > 0.05). All other factors being equal, each ‘best model’ selected was that with the strongest pseudo *R*-square statistics.

Results were regarded as significant at the 95% level (two tailed *p* < 0.05).

Some important relationships of confounding variables and well-being outcomes were estimated by unconditional logistic regression. Those symptoms experienced at least weekly over the preceding month we refer to as regular, three or more days weekly as frequent, and five or more days weekly as chronic.

## Results

There were 373 participants: 207 male (55.5%), 165 female (44.2%) and 1 transgender (0.3%). The participation rate was 85%. One invited student chose not to participate; the remaining non-participants did not have parental approval. The mean age was 12.3 years, ranging from 10.4 years to 13.7 years. We collected age, SES, and gender from all participants. Between 0.3% and 6.4% of data were missing from questions related to phone use. Only 139 (37%) WiFi responses were returned by parents. Parental responses specific enough to allow us to obtain cordless phone frequency and modulation system were received from 41% and 37%, respectively.

More than three-quarters (n = 285, 76.4%) of participants owned a cell phone (23 of these owned two). A further 12.8% reported regularly using someone else’s. Most (91%) participants reported using a cordless phone at home, and 47.5% had a wired landline (10% did not respond).

There were analogue and digital cordless phones, the latter utilising DECT, DECT6, WDECT, DSS, and FHSS modulation systems, each determining the way the signal is delivered in terms of radiofrequency and/or time. We categorised these in four groups: A) non-users of cordless phone B) analogue, C) DECT and DECT6, and D) the remainder, which all used a spread spectrum approach, most with randomised frequency hopping. The frequency ranges were A. 30-40 MHz and 900 MHz, B. 1.8 and 1.9 GHz, C.2.4 GHz, and D. 5.8 GHz. We grouped the first these as indicated to provide sufficiently large categories. Where a cross-tabulation indicated only one frequency was likely to be significant, the others were grouped as one category. Particular frequencies are not exclusive to each type of modulation system (Table 1).

**Table 1:** Frequency bands at which New Zealand cordless phone modulation systems operate

**Frequency**	**Modulation system**
30-40 MHz	Analogue
900 MHz	Analogue, DECT, DSS,
1.8-1.9 GHz	DECT, DECT 6, W-DECT
2.4 GHz	W-DECT, DSS
5.8 GHz	DSS, FHSS,

Cellphone modulation/systems included 1G, 2G and 3G. Their operating frequencies proved unhelpful to this study as so many utilised more than one frequency-band and switched randomly between them.

### Symptoms

Ordinal regression results of self-reported wireless phone use and well-being outcomes are presented in Tables 2, 3, 4, 5, 6, 7, 8. Statistically significant associations were detected in 31 of 86 models (36%).

**Table 2:** Associations between RF-EMF exposures and ‘Headache’ (symptom)

**Symptom**	**Symptom categories (N for unadjusted model/N for adjusted model)**	**Exposure**	**Exposure categories (N for unadjusted model/N for adjusted model)**	**Unadjusted OR (95% CI) for each exposure category**	**Adjusted OR (95% CI) for each exposure category**	**Confounders in model**
Headache	No, hardly ever (204/194)	Cordless calls > 10 mins. made and rec’d weekly	0 (64/59)	1	1	Cold/flu*+
	1 or 2 weekly (87/84)		1-2 (104/103)	1.27 (0.90-1.77)	1.28 (0.90-1.83)	**Woken by phone+**
	3 or 4 weekly (25/24)		3-9 (84/78)	1.40 (0.98-1.98)	1.35 (0.92-1.96)	Light out time
	Most days (16/15)		10-120 (80/77)	1.76 (1.23-2.51)*	1.41 (0.95-2.09)	
Headache	No, hardly ever (216/216)	Minutes on cordless phone daily	0-4 (148/148)	1	1	Cold/flu**+
	1 or 2 weekly (89/89)		5-15 (104/104)	0.83 (0.49-1.41**)**	0.78 (0.45-1.32)	
	3 or more times weekly (43/43)		16-240 (96/96)	**1.88 (1.14-3.12)**	**1.74 (1.05-2.90)**	
Headache	No, hardly ever (218/213)	Cellphone calls >10 minutes weekly	0 (277/271)	1	1	Cold/flu**+
	1 or 2 times weekly (91/91)		1-6 (66/66)	1.27 (0.95-1.69)	1.14 (0.84-1.53)	Woken by phone*+
	3 or 4 times weekly (29/29)		7-35 (12/12)	**2.37 (1.21-4.62)**	**2.40 (1.19-4.83)**	
	Most days (17/16)					
Headache	No, hardly ever (-/215)	Cellphone headset	No (320)	Model invalid	1	Cold/flu**+
	1 or 2 weekly (-/91)		Wired (16)		1.29 (0.71-2.36)	Woken by phone
	3 or more weekly (-/43)		Wireless (13)		**2.23 (1.10-4.53)**	Light out time
Headache	No, hardly ever (-/93)	Cordless phone frequency	Not exposed (-/28)	Model invalid	1	Cold/flu**+
	1 or 2 weekly (-/38)		≤ 900 MHz (-/19)		1.04 (0.38-2.90)	Woken by phone
	3 or more weekly (-/14)		1.8-1.9 GHz (-/27)		1.42 (0.61-3.34)	
			2.4 GHz (-/53)		0.74 (0.34-1.63)	
			5.8 GHz (-/18)		1.55 (0.61-3.96)	

**Bold font** Sig. *p* < 0.05; *Sig. *p* ≤ 0.01; **Sig. *p* ≤ 0.001; + positive association; ‘Model invalid’ means it did not meet the criteria outlined in the methods.

**Table 3:** Associations between RF-EMF exposures and ‘Tinnitus’ (symptom)

**Symptom**	**Symptom categories (N for unadjusted model/N for adjusted model)**	**Exposure**	**Exposure categories (N for unadjusted model/N for adjusted model)**	**UnadjustedOR (95% CI) for each exposure category**	**Adjusted OR (95% CI) for each exposure category**	**Confounders in model**
Tinnitus	No, hardly ever (-/194)	Cordless calls >10 minutes weekly	0 (-/61)	Model invalid	1	Cold/flu**+
	1 or 2 times weekly (-/73)		1-2 (-/102)		1.05 (0.75-1.49)	Use headset for calls*+
	3 or 4 times weekly (-/27)		3-9 (-/79)		0.70 (0.48-1.01)	Decile (SES)
	Most of the time (-/27))		10-120 (-/79)		1.05 (0.73-1.52)	
Tinnitus	No, hardly ever (-/207)	Minutes on cordless phone daily	0-4 (-/141)	Model invalid	1	**Cold/flu+**
	1-4 times weekly (-/101)		5-15 (-/100)		0.86 (0.64-1.13)	**Decile-**
	Most days (-/28)		16-240 (-/95)		1.03 (0.76-1.39)	Woken by phone
						Use headset for calls
Tinnitus‡	No, hardly ever (-/84 )	Minutes on cordless phone daily	0-4 (-/72)	Model invalid	1	Cold/flu*+
	1 or 2 times weekly (-/35)		5-15 (-/45)		0.60 (0.25-1.42)	Use headset for calls*
	3 or 4 times weekly (-/8)	*including*	16-240 (-/21)		0.96 (0.33-2.76)	**Cordless phone frequency**
	Most of the time (-/11)					
		Cordless phone frequency	Not exposed		1	Age
			≤ 900 MHz		**7.13 (1.5-33.7)**	
			1.8-1.9 GHz		7.32 (1.9-28.4)*	
			2.4 GHz		3.56 (0.95-13.4)	
			5.8 GHz		4.39 (0.99-19.5)	
Tinnitus	No, hardly ever (-/88)	Cordless phone frequency	Not exposed (30/27)	Model invalid	1	Cold/flu*+
	1 or 2 times weekly (-/36)		≤ 900 MHz (20/19)		**4.10 (1.03-16.28)**	**Use cellphone headset+**
	3 or 4 times weekly (-/9)		1.8-1.9 GHz (28/27)		**4.70 (1.34-16.43)**	
	Most of the time (12/11)		2.4 GHz (54/53)		2.46 (0.77-7.86)	
			5.8 GHz (18/18)		3.83 (0.99-14.82)	
Tinnitus	No, hardly ever (217/213)	Cellphone calls >10 minutes weekly	0 (275/270)	1	1	Cold/flu*+
	1 or 2 times weekly (79/77)		1-6 (65/65)	1.24 (0.92-1.66)	1.21 (0.90-1.64)	**Use of earpiece+**
	3 or 4 times weekly (26/26)		7-35 (12/11)	**2.00 (1.01-3.94)**	1.56 (0.73-3.35)	Decile-
	Most of the time (30/30)					
Tinnitus	No, hardly ever (-/220)	Cellphone headset	No (-/327)	Model invalid	1	Cold/flu*+
	1 or 2 times weekly (-/79)		Wired (-/16)		**1.80 (1.00-3.26)**	Decile
	3 or 4 times weekly (-/27)		Wireless (-/13)		1.61 (0.85-3.05)	
	Most of the time (-/30)					

**Bold font** Sig. *p* < 0.05; *Sig. *p* ≤ 0.01; **Sig. *p* ≤ 0.001; + positive association; - negative association; ‡ Previous model recalculated including cordless phone frequency (see text); ‘Model invalid’ means it did not meet the criteria outlined in the methods.

**Table 4:** Associations between RF-EMF exposures and feeling ‘Down or depressed’ (symptom)

**Symptom**	**Symptom categories (N for unadjusted model/N for adjusted model)**	**Exposure**	**Exposure categories (N for unadjusted model/N for adjusted model)**	**Unadjusted OR (95% CI) for each exposure category**	**Adjusted OR (95% CI) for each exposure category**	**Confounders in model**
Down/depressed	No (-/220)	Minutes on cordless phone daily	0- < 5 (-/141)	Model invalid	1	**Cold/flu+**
	Sometimes (-/85)		5-15 (-/100)		1.00 (0.75-1.37)	**Times woken by phone+**
	Often/All the time (-/32)		>15-240 (96)		1.02 (0.76-1.37)	Wireless earpiece
						Age
Down/depressed	No (-/206)	Cordless calls >10 minutes weekly	0-1 (-/108)	Model invalid	1	**Cold/flu+**
	Sometimes (-/85)		>1-5 (-/116)		0.92 (0.69-1.24)	**Times woken by phone+**
	Often/All the time (-/30)		>5-120 (-/97)		1.06 (0.77-1.46)	Wireless headset
						Age
Down/depressed	No (-/226)	Cellphone calls >10 minutes weekly	0 (-/269)	Model invalid	Model invalid	**Cold/flu+**
	Sometimes (-/87)		1-6 (-/66)			**Times woken by phone+**
	Often/All the time (-/33)		7-35 (-/11)			Wireless headset
						Age
Down/depressed	No (235/233)	Cellphone headset	No (328/327)	1	1	Cold/flu*+
	Yes, sometimes (90/90)		Wired (16/16)	1.09 (0.64-1.86)	0.90 (0.51-1.58)	**Times woken by phone+**
	Yes, often (27/27)		Wireless (14/13)	**1.96 (1.10-3.51)**	**2.04 (1.09-3.82)**	
	Yes, all the time (6/6)					
Down/depressed	No (99/98)	Cordless phone frequency	Not exposed (29/27)	1	1	**Cold/flu**
	Sometimes (38/35)		≤ 900 MHz (21/20)	**2.22 (1.13-4.38)**	**2.40 (1.15-5.02)**	Earpiece
	Often (11/10)		Other frequencies	1.28 (0.75-2.19)	1.38 (0.76-2.51)	
	All the time (3/3)		(101/99)			

**Bold font** Sig. *p* < 0.05; *Sig. *p* ≤ 0.01; + positive association; - negative association; ‘Model invalid’ means it did not meet the criteria outlined in the methods.

**Table 5:** Associations between RF-EMF exposures and ‘Wake up in the night’ (symptom)

**Symptom**	**Symptom categories (N for unadjusted model/N for adjusted model)**	**Exposure**	**Exposure categories (N for unadjusted model/N for adjusted model)**	**Unadjusted OR (95% CI) for each exposure category**	**Adjusted OR (95% CI) for each exposure category**	**Confounders in model**
Wake in the night	No, hardly ever (161/157)	Cordless calls >10 minutes weekly	0 (63/61)	1	1	Age*-
	1 or 2 weekly (110/107)		1-2 (103/102)	1.04 (0.74, 1.45)	1.10 (0.78, 1.56)	Woken by cellphone*+
	3 or 4 weekly (27/26)		3-9 (84/79)	1.07 (0.97, 1.53)	1.05 (0.73, 1.51)	Wireless headset
	Most days (32/30)		10-120 ( 80/78)	**1.50 (1.05, 2.16)**	1.40 (0.96, 2.04)	
Wake in the night	No, hardly ever (-/168)	Minutes on	0-4 (-/142)	Model invalid	1	Woken by phone**+
	1-2 times weekly (-/111)	cordless phone	5-15 (-/99)		1.26 (0.87, 1.81)	**Sex + ♀**
	3+ times weekly (-/57)	daily	16-240 (-/95)		0.82 (0.55, 1.23)	Cold/flu
Wake in the night	No, hardly ever (175/170)	Cellphone calls	0 (276/269 )	1	1	Woken by phone**+
	1 or 2 times weekly (112/112)	>10 minutes	1-6 (65/65)	**1.70 (1.07, 2.71)**	1.58 (0.98, 2.56)	Sex
	3 or 4 times weekly (31/30)	weekly	7-35 (12/12)	1.16 (0.47, 2.84)	0.85 (0.30, 2.36)	Cold/flu
	Most of the time (35/34)					
Wake in the night	No, hardly ever (178/175)	Cellphone headset	No (328/326)	1	1	Woken by phone**+
	1 or 2 times weekly (115/115)		Wired (16/16)	0.66 (0.29, 1.50)	0.65 (0.28, 1.49)	Cold/flu
	3 or more times weekly (30/30)		Wireless (13/12)	**2.24 (1.17, 4.29)**	**2.42 (1.21, 4.84)**	Age
	Most nights (34/34)					Sex
Wake in the night	No, hardly ever (71/70)	WiFi at home	No (70/68)	1	**1**	Woken by phone
	1 – 4 times weekly (52/50)		Yes (68/66)	**0.65 (0.45-0.63)**	**0.66 (0.44-0.99)**	Age
	Most nights (15/14)					Sex
						Cold/flu
						Wireless headset

**Bold font** Sig. *p* < 0.05; *Sig. *p* ≤ 0.01; **Sig. *p* ≤ 0.001; + positive association; - negative association; ♀female; ‘Model invalid’ means it did not meet the criteria outlined in the methods.

**Table 6:** Associations between RF-EMF exposures and having ‘Trouble falling asleep’ (symptom)

**Symptom**	**Symptom categories (N for unadjusted model/N for adjusted model)**	**Exposure**	**Exposure categories (N for unadjusted model/N for adjusted model)**	**Unadjusted OR (95% CI) for each exposure category**	**Adjusted OR (95% CI) for each exposure category**	**Confounders in model**
Trouble falling asleep	No (165/156)	Cordless calls > 10 minutes weekly	0-1 (111/105)	1	1	Time of lights out*+
	1 or 2 times weekly (88/85)		>1-5 120/114)	1.54 (0.54-2.53)	1.56 (0.93-2.62)	Sex
	3 or 4 times weekly (39/36)		>5-120 (99/95)	1.99 (1.19-3.34)*	1.58 (0.89-2.81)	Decile
	Most nights (38/37)					Times woken in night
Trouble falling asleep	No, hardly ever (184/183)	Minutes on cordless phone daily	0-4 (147/147)	1	1	Sex* + ♀
	1 or 2 times weekly (91/91)		5-15 (106/105)	1.14 (0.77-1.69)	1.00 (0.65-1.54)	**Decile+**
	3 or 4 times weekly (36/36)		16-240 (96/96)	**1.61 (1.06-2.43)**	1.41 (0.89-2.23)	Cold/flu
	Most nights (38/38)					
Trouble falling asleep	No, hardly ever (-/175)	Cellphone calls >10 minutes weekly	0 (-/267)	Model invalid	1	Woken by phone**+
	1 or 2 times weekly (-/90)		1-6 (-/63)		0.92 (0.67-1.25)	**Light out time+**
	3 or 4 times weekly (-/37)		7-35 (-/12)		1.38 (0.66-2.91)	**Decile+**
	Most nights (-/40)					Sex + ♀
Trouble falling asleep	No, hardly ever (-/179)	Cellphone headset	No (320)	Model invalid	1	Sex** + ♀
	1 or 2 times weekly (-/92)		Wired (16)		0.41 (0.16-1.08)	Light out time*+
	3 or more times weekly (-/77)		Wireless (12)		1.23 (0.51-2.97)	**Decile+**
						Age
						Times woken

**Bold font** Sig. *p* < 0.05; *Sig. *p* ≤ 0.01; **Sig. *p* ≤ 0.001; + positive association; ♀female; ‘Model invalid’ means it did not meet the criteria outlined in the methods.

**Table 7:** Associations between RF-EMF exposures and being ‘Tired at school’ (symptom)

**Symptom**	**Symptom categories (N for unadjusted model/N for adjusted model)**	**Exposure**	**Exposure categories (N for unadjusted model/N for adjusted model)**	**Unadjusted OR (95% CI) for each exposure category**	**Adjusted OR (95% CI) for each exposure category**	**Confounders in model**
Tired at school	No, hardly ever (-//61)	Cordless calls >10 minutes weekly	0 (-/60)	Model invalid	1	Woken by phone**+
	1 or 2 weekly (-/153)		1-2 (-/101)		1.17 (0.81-1. 69)	**Age-**
	3 or 4 weekly (-/41)		3-9 (-/80)		0.73 (0.50-1.07)	Cold/flu
	Most days (-/64)		10-120 ( -/78)		0.90 (0.60-1.34)	
Tired at school	No, hardly ever (-/73)	Minutes on cordless phone daily	0-4 (-/137)	Model invalid	1	Woken by phone **+
	1-2 times weekly (-/144)		5-15 (-/100)		0.82 (0.86-1.14)	Cold/flu*+
	3+ times weekly (-/114)		16-240 (-/94)		0.79 (0.55-1.12)	Light out time
Tired at school	No, hardly ever (-/74)	Cellphone calls >10 minutes weekly	0 (-/270)	Model invalid	1	Woken by phone**+
	1 or 2 times weekly (-/155)		1-6 (-/65)		1.26 (0.75-2.10)	**Cold/flu+**
	3 or 4 times weekly (-/46)		7-35 (-/12)		0.72 (0.23-2.25)	**Decile+**
	Most of the time (-/72)					
Tired at school	No (-/34)	Cordless phone frequency	Not exposed (-/26)	Model invalid	1	Cold/flu*+
	1-4 days weekly (-/78)		≤ 900 MHz (-/20)		1.26 (0.59-2.70)	**Light out time +**
	Most days (-/30)		1.8-1.9 GHz (-/25)		1.92 (0.93-3.96)	Use phone headset
			2.4 GHz (-/53)		1.01 (0.55-1.87)	Woken at night
			5.8 GHz (-/18)		1.79 (0.83-3.87)	TV in bedroom
Tired at school	No, hardly ever (35/32)	Cordless phone system	Not exposed (29/25)	1	1	**Light out time +**
	1-4 days weekly (75/71)		DECT (51/50)	1.30 (0.73-2.33)	1.09 (0.57-2.11)	Use phone headset
	Most days (27/26)		DSS (27/25)	2.50 (1.30-4.80)*	2.53 (1.21-5.31)*	Woken at night
			Analogue (30/29)	1.51 (0.80-2.87)	1.24 (0.60-2.58)	Cold/flu
						TV in bedroom
Tired at school	No, hardly ever (-/75)	Cellphone headset	No (-/318)	Model invalid	1	Woken by phone**+
	1 or 2 times weekly (-/155)		Wired (-/16)		1.11 (0.42, 2.93)	Cold/flu*+
	3 or 4 times weekly (-/47)		Wireless (-/13)		1.38 (0.47, 4.04)	**Decile+**
	Most days (-/70)					Light out time

**Bold font** Sig. *p* < 0.05; *Sig. *p* ≤ 0.01; **Sig. *p* ≤ 0.001; + positive association; - negative association; ‘Model invalid’ means it did not meet the criteria outlined in the methods.

**Table 8:** Associations between RF-EMF exposures and having a ‘Painful texting thumb’ (symptom)

**Symptom**	**Symptom categories (N for unadjusted model/N for adjusted model)**	**Exposure**	**Exposure categories (N for unadjusted model/N for adjusted model)**	**Unadjusted OR (95% CI) for each exposure category**	**Adjusted OR (95% CI) for each exposure category**	**Confounders in model**
Painful texting thumb	No (268/212)	Cordless calls > 10 minutes weekly	0 (62/46)	1	1	**Cold/flu+**
	1 or 2 times weekly (44/40)		1-2 (103/84)	4.11 (1.16-14.54)*	3.65 (0.99-13.53)	Sex
	3 times to most (17/17)		3-9 (84/65)	**3.88 (1.90-14.11)**	2.54 (0.66-9.83)	Age
			10-120 (80/74)	9.61 (2.77-33.42)**	7.02 (1.93-25.53)*	
Painful texting thumb	No, hardly ever (287/287)	Minutes on cordless phone daily	0- < 5 (147/147)	1	1	**Cold/flu+**
	1 or 2 weekly (42/42)		5-15 (103/103)	**2.34 (1.17-4.71)**	**2.26 (1.12-4.54)**	
	3 or more times weekly (17/17)		<15-240 (96/96)	3.37 (1.73-6.57)**	4.54 (1.62-6.19)**	
Painful texting thumb	No, hardly ever (-/287)	Cellphone calls >10 minutes weekly	0 (-/274)	Model invalid	1	**Cold/flu+**
	1 or 2 times weekly (-/45)		1-6 (-/65)		1.37 (0.70-2.70)	**Sex- ♂**
	3 or more times weekly (-/19)		7-35 (-/12)		**4.43 (1.27-15.44)**	Age
Painful texting thumb	No (157/157)	Billed texts sent weekly	0 (42/42)	1	1	**Cold/flu+**
	1 or 2 times weekly (27/27)		1-99 (74/74)	7.55 (0.96-59.3)	6.39 (0.80-51.2)	TV in bedroom
	3 to most of the time (10/10)		100-499 (63/63)	**16.46 (2.14**-126.3)*	**11.94 (1.52**-94.1)*	Age
			500+ (15/15)	**27.19 (2.99**-247.4)*	**21.54 (2.24**-**207.5)**	

**Bold font** Sig. *p* < 0.05; *Sig. *p* ≤ 0.01; **Sig. *p* ≤ 0.001; + positive association; - negative association; ♂male; ‘Model invalid’ means it did not meet the criteria outlined in the methods.

Few participants chose not to answer the well-being questions (headache: 1; down/depressed, trouble falling asleep, wake in the night: 3 each; tinnitus, painful thumb, tired at school: 4 each).

There were several positive dose-dependent relationships. This applied to all well-being outcomes except ‘waking in the night’ and ‘tired at school’, although not all were statistically significant and some lost that significance once other influences were accounted for.

One of the most consistent associations with time spent on a cellphone or cordless phone was the prevalence of headaches (Table 2), specifically, minutes of cordless use daily (OR 1.74, CI 1.05, 2.90) and the number of long cellphone calls weekly (OR 2.40, CI 1.19, 4.83).

Associations between the extent of cordless/cellphone use and tinnitus (Table 3) or depression (Table 4) were not sufficiently consistent to draw any clear conclusions. However, tinnitus was statistically significantly related to the use of a wired cellphone headset (OR 1.80, CI 1.00, 3.26).

Indeed the use of cellphone headsets was one exposure type most consistently associated with well-being outcomes. Use of a wireless headset was related to headache prevalence (OR 2.23, CI 1.10, 4.53), feeling down/depressed (2.04, CI 1.09, 3.82), and waking in the night (OR 2.42, CI 1.21, 4.84) (Table 5). It should be noted that there were rather few participants who used one; despite this the confidence intervals are reasonable.

Having trouble falling asleep (Table 6) had a dose-dependent relationship with cordless phone use (>15 minutes daily OR 1.61 (1.06-2.43) and > 5 long calls weekly OR1.99 (1.19-3.34), but these lost statistical significance in adjusted models.

Tiredness at school (Table 7) was not significantly associated with the assessed RF-EMF exposures.

Tinnitus and feeling down or depressed were associated with specific cordless phone radiofrequency bands but not to types of modulation.

The only statistically significant negative relationship was a reduced likelihood of waking in the night for those with WiFi at home (OR 0.66 CI 0.44-0.99).

There were strong association between having a painful thumb and texting as well as wireless phone use (Table 8). They are discussed below.

Several confounding variables were related to well-being outcomes (Table 9). Many participants reported having headaches at least weekly (37.5%) over the last month. This was associated with having a cold or flu in that period. The data collection took place in early- to mid-winter during an influenza pandemic, perhaps explaining 59% having had a cold or flu within the month before the survey leading to this variable being an important consideration in the analyses.

**Table 9:** **Sample relationships of confounding variables and well**-**being outcomes estimated by unconditional logistic regression**

**Confounding variable**	**Symptom**	**N**	**OR (95% CI)**
Cold/flu in last month	Headache^a^	370	**2.50 (1.57**- **3.99)**
Cold/flu in last month	Down/depressed^b^	369	**2.38 (1.04**- **5.45)**
Cold/flu in last month	Tired at school^b^	368	**2.07 (1.29**- **3.32)**
Cold/flu in last month	Sore texting thumb^a^	368	**2.49 (1.31**- **4.75)**
Woken by cellphone at least weekly	Headache^b^	356	**4.70 (2.38**- **9.29)**
Woken by cellphone at least weekly	Headaches^c^	356	**5.89 (1.89**- **18.30)**
Woken by cellphone at least weekly	Down/depressed^b^	355	**2.42 (1.14**- **5.12)**
Woken by cellphone at least weekly	Tinnitus^b^	355	**2.46 (1.33**- **4.56)**
Woken by cellphone at least weekly	Tired during school^d^	354	**3.49 (1.97- 6.2)**
SES^d^	Tired during school^a^	368	
Low			**0.40 (0.18, 0.87)**
Mid			0.59 (0.34, 1.01)
High			1
SES^d^	Tinnitus^c^	368	
Low			1
Medium			**0.17 (0.05, 0.57)**
High			0.41 (0.15, 1.08)
Sex	Trouble falling asleep^a^	369	
Boys			1
Girls			**1.97 (1.29, 3.00)**
Sex	Wake up in the night^a^	369	
Boys			1
Girls			**1.57 (1.04, 2.38)**
Sex	Tired during school^c^	368	
Boys			1
Girls			**0.51 (0.30, 0.88)**
Time of light out, per minute after 7 pm	Trouble falling asleep^b^	363	**1.008 (1.003, 1.012)**
Phone location at night	Headache^a^	361	
Another room			1
By bed			**1.95 (1.20, 3.15)**
Under pillow			0.98 (0.43, 2.22)
Distance from eyes for bed-texting	Headache^a^		0.96 (0.93, 0.99)

^a^Regular; ^b^Frequent; ^c^Chronic; ^d^Socioeconomic status according to school decile grouping.

Each model controls for sex, age, and SES, and is estimated by unconditional logistic regression.

Tinnitus and feeling down or depressed were also common (38% and 35%, respectively) at least weekly, as were tiredness at school (77%), trouble falling asleep (48%) and waking in the night (50%).

Logistic regression indicated a strong positive association between being woken in the night by the cellphone and being tired at school, with the odds of being tired most school days being OR 3.49 (1.97-6.2).

## Discussion

This study found many significant associations between the reduction in young adolescents’ self-reported general well-being and their extent of exposure to RF-emitting technology. This 36% of the ordinal logistic regression analyses is more than could be expected by chance alone (5%).

The symptoms most consistently related to the tested RF-EMF exposures were headache and, surprisingly, having a painful thumb.

### Headache

The duration and number of cordless and cellphone calls consistently indicated an increased, dose-dependent risk of suffering from headache. A possible explanation for the apparent link between headache and cellphone use was given by Frey who noted the involvement of the blood-brain barrier and the dopamine-opioid systems of the brain in headaches, both of which have been linked to RF-EMF exposures similar to those from cellphones [12]. An increased prevalence of headache with wireless phone use is the most consistently reported well-being association [13-15]. The increased risk reported by Söderqvist et al. [15] in a study of 15 to 19 year-olds was very similar to that demonstrated for cordless phone use here. That study also assessed depression, tinnitus and sleep disturbance; their results from more than 15 minutes daily cellphone use were similar in each case to those of more than 15 minutes daily cordless phone use in this study.

### Tinnitus and depression

Only six participants in our study reported feeling chronically down or depressed. We note, though, that prevalence was tenfold higher in those who had a wireless headset. Although tinnitus and depression were not significantly related to wireless phone calls after allowing for common confounders, this was not the case with relation to the operating frequency. Those whose home cordless phones operated on ≤ 900 MHz, 1.8-1.9 GHz, and 5.8 GHz (the latter fell just below the 95% significance threshold), and those who used a wired headset with their cellphone, were at a greater risk of having tinnitus. We wondered whether these associations could have a smoothing effect on our modelled results for cordless phone use, so tested a model for tinnitus incorporating both. This approach reduced the odds of tinnitus from using *any* cordless phone, but considerably increased the odds for those using one operating on one of the bandwidths that we had already identified as significantly related, as well as for those using a wired headset for calls (Table 3). Tinnitus is associated with elevated intracellular calcium levels and local oxidative stress which are expected to affect the cochlea in the inner ear [16]. Intracellular calcium levels are affected by electromagnetic fields dependent upon specific frequency ‘windows’ [17,18] and there have been reports of oxidative stress induced in mitochondrial DNA of cortical neurons by exposure to 1.8 GHz cellphone exposure [19].

We found that those who used a wireless headset, on the other hand, were at more risk of experiencing headaches, feeling down or depressed, or waking in the night.

There are possible explanations for some of these outcomes other than RF-EMF exposure. It could be that students with wired headsets for their cellphones were also more likely than those with wireless (or no) headset to listen to music this way, and that the tinnitus was instead related to the volume or duration of music listened to by the affected students. However, it is harder to find alternative explanations for specific frequency effects. For instance, it is possible that those who felt down or depressed responded by spending longer on the cordless phone, but this does not explain why some radiofrequencies would have a higher association with depression than others.

### Painful thumb

As we were able to retrieve billing data on the extent of texting, we asked about whether students experienced pain in the thumb they used for texting. Perhaps not surprisingly, there was a positive relationship. However it was unexpected to also find a strong relationship with wireless phone use. The extent of use of both phone types was related to having a sore thumb, with a higher risk related to lengthy cordless calls than to the number of texts sent. If the pain had only been related to texting it would have suggested a simple mechanical effect similar to Occupational Overuse Syndrome, but no such action is involved in lengthy calls. The cause could not be identified in this study.

### Sleep and sleepiness

We found a strong link between being woken by the cellphone and both tiredness at school and headache prevalence, and therefore took account of this in adjusted models for waking in the night. This relationship is not related to RF-EMF exposure, but will have important consequences. Daytime tiredness and headache are both likely to impact negatively on students’ ability to learn effectively at school. Storing cellphones away from bedrooms overnight would remove this source as a reason for broken sleep.

Daytime sleepiness (fatigue), while not appearing to be related to wireless phone use, had significant results for those using a Digital Spread Spectrum (DSS) cordless system. Because daytime sleepiness was related to phone’s form of transmission but not phone use, it suggested that the responsible RF-EMFs may be those constantly emitted from the cordless phone base, not the handset. If effects do differ with either frequency band or transmission system, this may explain conflicting results as until now studies that have included cordless phone exposure at all have not accounted for their different operating frequencies and modulations. The finding is preliminary and further research is needed to confirm it.

### WiFi

The only well-being indicator significantly related to having WiFi at home was a reduced likelihood of waking in the night 0.66 (0.44-0.99). This was also the only significant negative relationship, although long cellphone calls and daily time spent on a cordless phone were similar. At the time of the survey, New Zealand home WiFi systems operated in the 2.4 GHz (the default setting) or 5.8 GHz bandwidths, using a frequency-hopping modulation system. A review published in 2004 concluded that, “there seems to be some consistency regarding a slight sleep-promoting effect and an increase of the alpha power of the sleep EEG induced by high-frequency EMFs” [20]. Several of the reviewed studies included normally encountered very low frequencies. WiFi systems incorporate a beacon frame transmission resulting in ≈ 10 Hz extra low frequency [21]. 10 Hz falls within the alpha range of brain activity, typical of the transition from waking to sleep, but not the reverse, cited in Hung et al. [22].

This variable had a low response (37%) and the distribution is not properly representative of the whole group, so this is a tentative result.

### Personal dosimeters versus self-reporting

Both self-reported extent of phone use and measured levels of personal exposure have disadvantages. Body-worn dosimeters are likely to underestimate daytime exposure due to the influence of the body’s own electric activity and night-time measurements are not a valid proxy of exposure [23]. On the other hand, estimated phone use, and related RF-EMF exposure, has been criticised as unreliable due to inaccuracies of estimation [24]. Even with a full and accurate record of the use of wireless equipment, it is impossible to accurately know the amount of energy absorbed due to the many factors that affect this. We believe that the use of both dosimeters and self-reporting are valid in helping construct a picture of wireless phone wellbeing associations [25].

### Strengths and limitations

Our study had several strengths. The sample was representative of the region and there was a high response-rate among the adolescents. Data collection and entry were carried out by the first author thereby eliminating inter-rater-error.

There were some limitations. Using school decile as a surrogate for SES will have resulted in some misclassification of individual SES. Some variables had low parental responses: cordless phone frequency (n = 152), transmission system (n = 138), and WiFi (n = 139), resulting in a skewed SES representation.

We doubt the ‘nocebo effect’ or knowledge that well-being questions were being asked impacted on the reported extent of phone use the following reasons. The well-being questions were given a low profile and introduced last. Participants were invited to comment on the well-being questions if they wished. No-one indicated a belief that RF-EMF exposure of any kind may be associated with their level of well-being or health, although a few had noticed a high pitch while near a television or when sitting at the computer. On the other hand, some gave responses such as, “The headaches and tiredness has nothing to do with my cell phone” and “this is not by using a phone”. It is plausible that these attitudes affected the well-being responses of some as indicated by one participant who assigned the lowest category to all well-being questions because “if I get them they’re not related to cellphone use.” An exception would be the phrase ‘Painful texting thumb’, which may have affected responses.

We did not ask about existing medical conditions or medications. Neither did we ask about the distance of the cordless phone base from the bed. Future research assessing effects from cordless phone exposure should adjust for this.

## Conclusions

Young people who made heavier use of cordless and cell phones reported an increased prevalence of headache. This was not the case overall for tinnitus or depression after allowing for common confounders. However those who had cordless phones operating on some frequency bands or modulation system, and those who used a headset with their cellphone were at a greater risk of suffering these conditions.

Use of a headset is commonly recommended to reduce RF-EMF exposure to the head during cellphone calls. Our results suggest that using speaker-phone instead would be better for calls over 15 minutes.

A new and possibly important finding was the apparent significance of some frequency bands or systems used by cordless phones. This may apply also to cellphones. Our findings suggest the need to explore further the effects of cordless phone protocols (operating frequency and modulation system). An advantage of examining cordless phone use is that individual phones almost exclusively employ only one frequency band and one modulation type, unlike cellphones which commonly use several frequency bands and more than one modulation depending upon circumstances such as data traffic or terrain. Further, cordless phones generally operate on full power making it easier to calculate the power output to which the user is exposed. Future research involving children’s health and well-being and exposure to RF-EMFs should include cordless phone exposure – particularly considering frequency and modulation.

Passive exposure at home can be reduced substantially by placing cordless phone bases and WiFi routers in an area of the house remote from the bedrooms. To safeguard young people’s well-being, we suggest it would be prudent to restrict their use of cellphones and cordless phones to less than 15 minutes daily. If parents were to require cellphones, cordless phones and other RF-EMF transmitting devices not to be in bedrooms overnight, this would remove a source of RF-EMF exposure and remove the significant likelihood of calls or texts causing broken sleep, which we demonstrate was strongly related to tiredness at school.

## Abbreviations

RF: Radiofrequency; RF-EMF: Radiofrequency electromagnetic fields; OR: Odds ratio; CI: Confidence interval; DECT: Digital enhanced cordless telephone; 1G: 2G, 3G, 1^st^, 2^nd^ and 3^rd^ Generation; DECT6: Technology using the DECT modulation system but on the 1800 MHz band in NZ for less interference; WDECT: Wideband DECT incorporates frequency hopping to use a wider frequency band; DSS: Digital spread spectrum; FHSS: Frequency-hopping spread spectrum; SES: Socioeconomic status; WiFi: Wireless broadband; GSM: Global system for mobile communication.

## Competing interests

Mary Redmayne and Euan Smith have no competing interests to declare. Michael Abramson holds small parcels of shares in Telstra and SingTel which operate cell telephone networks in Australia. The study was not funded.

## Authors’ contributions

The study was designed by MR and MJA. MR conducted the survey analysed the data and wrote the first draft of the paper. MR and MJA analysed implications. All authors contributed to the paper and approved the final version for publication.
